# New Appliances and Things Medical

**Published:** 1893-07-01

**Authors:** 


### NEW APPLIANCES AND THINGS MEDICAL.

### MESSRS. ALLEN AND HANBURY'S PREPARATIONS.

We have received samples of Messrs. Allen and Hanbury's Bynin, a liquid preparation of malt of high diastasic power, yet being free from the thick, tenacious, treacle-like consistency, which patients of feeble digestive capacity dislike so extremely. The same firm has introduced several excellent and very useful and elegant preparations of liquid malt, such as Byno-Pepsin and Byno Pancreatine, combinations that are so frequently demanded in practice. Coca-Bynin combines the active principles of coca leaves with liquid malt, and is a pleasant and efficient mode of obtaining the stimulating properties of cocaine in minute doses, while aiding digestion and assimilation. We have pleasure in noticing the sample of the cod liver oil emulsion. Their perfected cod liver oil is in itself so free from disagreeable taste and smell that we often find an emulsion unnecessary, even for delicate digestions, but with this emulsion we can readily overcome the prejudices of the most fastidious patients. Of the gelatine-coated pills submitted to us we can speak most highly. They are unsurpassed in elegance and in ready solubility.

